# MicroRNA prediction based on 3D graphical representation of RNA secondary structures

**DOI:** 10.3906/biy-1904-59

**Published:** 2019-08-05

**Authors:** Müşerref Duygu SAÇAR DEMİRCİ

**Affiliations:** 1

**Keywords:** MicroRNA, RNA structure, machine learning, random forest, decision tree, naïve Bayes

## Abstract

MicroRNAs (miRNAs) are posttranscriptional regulators of gene expression. While a miRNA can target hundreds of messenger RNA (mRNAs), an mRNA can be targeted by different miRNAs, not to mention that a single miRNA might have various binding sites in an mRNA sequence. Therefore, it is quite involved to investigate miRNAs experimentally. Thus, machine learning (ML) is frequently used to overcome such challenges. The key parts of a ML analysis largely depend on the quality of input data and the capacity of the features describing the data. Previously, more than 1000 features were suggested for miRNAs. Here, it is shown that using 36 features representing the RNA secondary structure and its dynamic 3D graphical representation provides up to 98% accuracy values. In this study, a new approach for ML-based miRNA prediction is proposed. Thousands of models are generated through classification of known human miRNAs and pseudohairpins with 3 classifiers: decision tree, naïve Bayes, and random forest. Although the method is based on human data, the best model was able to correctly assign 96% of nonhuman hairpins from MirGeneDB, suggesting that this approach might be useful for the analysis of miRNAs from other species.

## 1. Introduction

Ribonucleic acid (RNA) is a major player in many cellular processes and for some organisms it is the source of genetic information. Not only the sequences but also the structures of the RNA molecules have great importance. There are three main levels of RNA structure: primary (base sequence), secondary (based on base pairs, e.g., hairpins or the cloverleaf structure of transfer RNA (tRNA)), and tertiary (interactions between secondary structure elements) (Batey et al., 1999).The RNA secondary structure is formed by hydrogen bonds between base pairs A-U and G-C (G-U pairing is often observed) (Varani and McClain, 2000). However, these bases and pairings do not have the same strength. The four bases can be divided into several classes, such as based on the strength of the hydrogen bond (weak H-bonds (A, U) and strong H-bonds (G, C)), based on the amino group (A, C) and keto group (G, U), and according to chemical structures of purine (A, G) and pyrimidine (C, U). By using the properties of bases and pairing information, various methods aiming to measure RNA similarity have been proposed. Some of these approaches are based on graphical representation of RNA 2D structure, which might suffer from the loss of information (Zhang et al., 2016). On the other hand, methods developed for 3D graphical representation of RNA secondary structures use sequence, chemical, and structural information. The method developed by Zhang et al. (2016) for dynamic 3D graphical representation for RNA structure analysis seems to be performing better than other approaches.In recent years, the small, noncoding RNAs known as microRNAs (miRNAs) that regulate posttranscriptional gene expression have been studied extensively. There are various reasons for miRNAs’ popularity. For instance, a wide range of organisms produce miRNAs and there are some reports about their involvement in host-parasite interactions (Saçar Demirci et al., 2016; Acar et al., 2018). Moreover, many disease phenotypes are associated with miRNAs, and it is possible to use miRNAs as disease markers and new therapeutic agents (Avci and Baran, 2014; Tüfekci et al., 2014). However, considering the capacity of a eukaryotic genome to produce miRNA precursors, it is a difficult task to distinguish new miRNAs experimentally. As a result, designing and employing computational approaches for miRNA analysis have become essential subjects.In addition to the capacity of single-stranded RNAs forming secondary structures by self-folding, miRNAs have a characteristic hairpin structure so that they can be recognized and modified by miRNA biogenesis machinery elements (Kozlowski et al., 2008). Thus, miRNA prediction analyses usually require information from primary and secondary structures. Unfortunately, this hairpin structure is not a unique property of miRNAs (Roden et al., 2017). The majority of tools designed to determine if a given sequence is miRNA are based on the application of machine learning (ML) (Saçar Demirci et al., 2017). Although ML is quite powerful and advantageous for miRNA studies, there are some essential points to consider for an efficient analysis such as data quality, feature selection, and ML algorithm selection (Saçar Demirci and Allmer, 2017a). In this paper, for the first time, a ML framework for miRNA prediction based on the 3D representation of known miRNA precursors and pseudohairpins is proposed. The method is developed and tested based on human miRNA data, but it is possible to apply and/or extend it for other organisms as well. 

## 2. Materials and methods

Identification of miRNA hairpins is usually achieved by using 2-class classification-based ML approaches. In order to create models and test the effect of these models, different datasets were obtained and various classification algorithms were used in a workflow system.

### 2.1. Data

Sequence datasets that were used in training and testing were as follows:- 1917 human precursors (miRBase Release 22) (Kozomara and Griffiths-Jones, 2014) - learning data- 587 human precursors (MirGeneDB 2.0) (Fromm et al., 2018) - learning data- 7701 nonhuman precursors (MirGeneDB 2.0) (Fromm et al., 2018) - testing data- 8492 pseudohairpins (Ng and Mishra, 2007) - learning data

### 2.2. 3D graphical representation of RNA secondary structures

**Figure 1:**
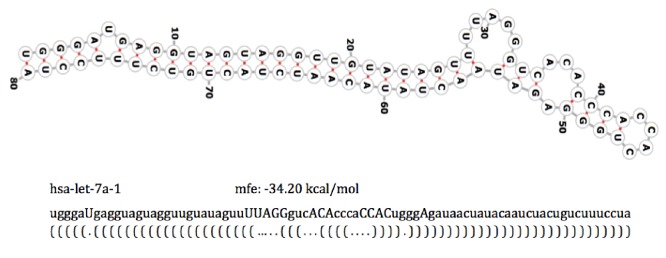
Representation of sequence and secondary structure of hsa-let-7a-1. mfe: Minimum free energy.

Secondary structures of RNA sequences were obtained by using RNAfold (Hofacker, 2003) with default settings. The best structure for each sequence was selected based on minimum free energy values (Figure  1). According to the dot-bracket (nonbonding and bonding bases, respectively) representation of 2D structures, bases in the sequence were modified as uppercase and lowercase characters. These sequences were then used as input to the RnaFeatureGenerator software (Zhang et al., 2016) to produce 36D vectors characterizing RNA secondary structures (Figure  1). 

### 2.3. Data mining

**Figure 2:**
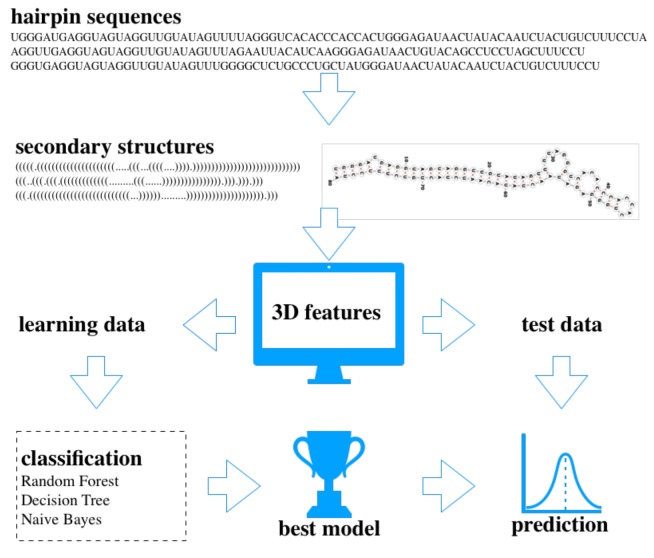
The basic workflow of the analysis. Hairpin sequences were folded into their secondary structures and based on the state of the bases (bonded or nonbonded) for each hairpin 3D features were calculated. Learning datasets were used for classification analysis with 1000-fold Monte Carlo cross-validation and the best models with the highest accuracy scores were applied to the test datasets for prediction.

The Konstanz Information Miner (KNIME) (Berthold et al., 2008) platform was used for the data mining analysis. Datasets containing 36D vectors were loaded and used for classification. For learning, 3 classifiers, random forest, decision tree, and naïve Bayes, were trained with human miRNAs from miRGeneDB as positive and pseudohairpins as negative examples (Figure  2). To avoid class imbalance, equally sized samples from both datasets were selected randomly, and 70% learning - 30% testing sets were applied with 1000-fold Monte Carlo cross-validation (Xu and Liang, 2001). The models from each classifier with the highest accuracy score were saved and used for further testing analysis. The same learning strategy was followed, where miRBase human precursor sequences were used as positive data. To test the performance of the model, nonhuman precursors from MirGeneDB were used. Datasets and the best models are available in the supplementary files (https://data.mendeley.com/datasets/dms72w9ckc/1). 

## 3. Results

**Figure 3:**
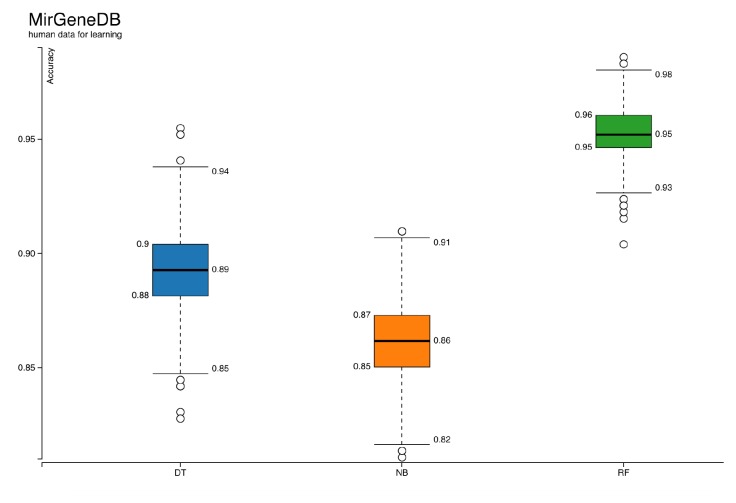
Accuracies of classifiers when positive dataset is MirGeneDB human precursors. DT: Decision tree, NB: naïve Bayes, RF: random forest.

**Figure 4:**
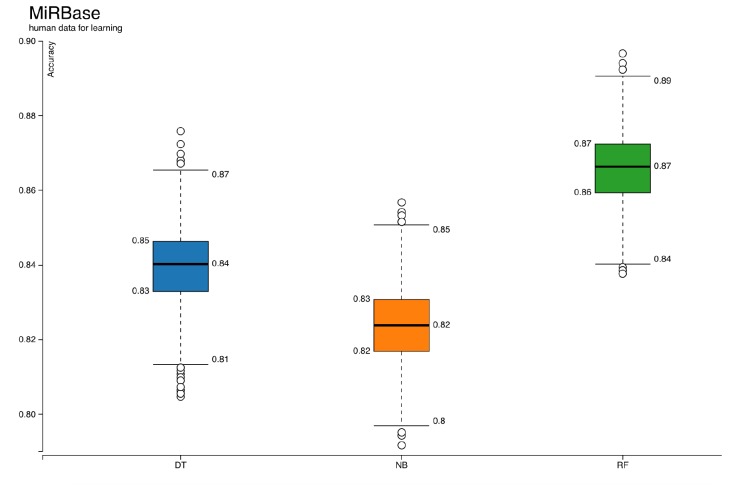
Accuracies of classifiers when positive dataset is miRBase human precursors. DT: Decision tree, NB: naïve Bayes, RF: random forest.

ML has been a popular choice for miRNA studies (Saçar Demirci et al., 2017). However, there are many factors affecting the performance of ML-based approaches (Saçar Demirci and Allmer, 2017a, 2017b). Here, not only a new workflow for miRNA precursor prediction is proposed, but also some crucial points for reliable results are investigated. For instance, the effect of positive dataset selection on the accuracy of learners is shown in Figures 3 and 4. The models trained with validated human miRNA sequences obtained from MiRGeneDB (Figure  3) have higher scores than models generated with miRBase (Figure  4). Graphs for other measures like precision, recall, specificity, sensitivity, and F-measure are provided in the supplementary files. Three classifiers were used simultaneously on the same datasets for learning. Among them, random forest showed better performance based on almost all of the measures (Figure  3 and  4 ; Supplementary Figures  1  and  2 ). Due to this clear difference, the random forest model with the highest accuracy score from Figure  3 was selected for further analysis. The next step was analyzing the capacity of the model on new datasets. According to results shown in Table 1and Supplementary Figure  3among 7701 miRNA precursors from 32 nonhuman species listed in MirGeneDB, around 4% were labeled as negative, meaning that even though the model was generated based on human data, it might also be applied to analyze miRNAs from other organisms. When the developed model was compared with some of the existing approaches using classification for miRNA prediction, as shown in Table 2results showed that the prediction accuracy of our method was greater than the Triplet-SVM, MiPred, MicroPred, and izMiR. The performance scores of sensitivity, specificity, and accuracy were taken from the articles of corresponding methods. Considering that all of these approaches were constructed by using differing parts such as types of classifiers, sampling methods, and datasets, comparison of their performances cannot be achieved based on their reported performance measurements. Nevertheless, the values are presented here to provide a general idea.

**Table 1:** Prediction results for other organisms’ miRNA hairpins. All organisms from MirGeneDB (except human) were included. MiRNA #shows total number of hairpins per species. Prediction column shows the number of miRNA and negative predictions, respectively. The table is sorted alphabetically for species.

Species	Acronym	MiRNA #	Prediction
Anolis carolinensis	Aca	261	244, 17
Alligator mississippiensis	Ami	272	259, 13
Ascaris suum	Asu	95	94, 1
Branchiostoma floridae	Bfl	90	88, 2
Bos taurus	Bta	433	418, 15
Caenorhabditis elegans	Cel	139	135, 4
Canis familiaris	Cfa	444	427, 17
Crassostrea gigas	Cgi	150	145, 5
Columba livia	Cli	246	237, 9
Chrysemys picta bellii	Cpi	290	278, 12
Cavia porcellus	Cpo	397	384, 13
Capitella teleta	Cte	102	96, 6
Drosophila melanogaster	Dme	152	133, 19
Dasypus novemcinctus	Dno	373	362, 11
Daphnia pulex	Dpu	79	67, 12
Danio rerio	Dre	385	369, 16
Eisenia fetida	Efe	192	177, 15
Echinops telfairi	Ete	339	328, 11
Gallus gallus	Gga	262	248, 14
Ixodes sp.	Isc	56	52, 4
Lottia gigantea	Lgi	80	79, 1
Macaca mulatta	Mml	498	488, 10
Mus musculus	Mmu	448	428, 20
Oryctolagus cuniculus	Ocu	366	361, 5
Ptychodera flava	Pfl	83	81, 2
Patiria miniata	Pmi	58	54, 4
Rattus norvegicus	Rno	413	394, 19
Sarcophilus harrissii	Sha	417	409, 8
Saccoglossus kowalevskii	Sko	83	80, 3
Strongylocentrotus purpuratus	Spu	57	51, 6
Tribolium castaneum	Tca	188	186, 2
Xenopus tropicalis	Xtr	253	241, 12

**Table 2:** Comparison of the model developed in this work with the existing classifiers. FN: Number of features used to build the classification model, ML: machine learning method, SE: sensitivity, SP: specificity, Acc: accuracy, SVM: support vector machine, NB: naïve Bayes, MLP: multilayered perceptron, RF: random forest, DT: decision tree.

Method	FN	ML	SE	SP	Acc
Triplet-SVM (Xue et al., 2005)	32	SVM			93.30
MiPred (Jiang et al., 2007)	34	RF, SVM	98.21	95.09	96.68
MicroPred (Batuwita et al., 2009)	21	RF, SVM	90.02	97.28	
izMiR (Saçar Demirci et al., 2017)	~900	SVM, NB, DT	91.98	91.98	91.25
3D model	36	RF, NB, DT	98.87	98.87	98.58

## 4. Discussion

The majority of tools developed for miRNA identification are based on ML; thus, they are affected by the challenges of ML. For instance, one of the most important criteria for a successful classification system is having high quality datasets (Saçar Demirci and Allmer, 2017b). For miRNA analysis, established miRNAs available in public databases, like miRBase and MirGeneDB, are used as positive data. Unfortunately, it is demanding to create a true negative dataset since it should have entries with similar characteristics to known miRNAs, but not too similar so that the algorithm can accurately discriminate between them. Hence, it is currently impossible to have a true validated negative dataset. The most popular negative dataset, known as pseudohairpins, was selected and used for this study. Not only negative datasets but also positive ones seem to need further improvement. Previously, it has been shown that some of the entries in miRBase are unlikely to be true miRNAs (Saçar et al., 2013). Moreover, the results presented here show that in terms of quality, human miRNAs listed in MirGeneDB are better than human miRNA entries in miRBase. Nevertheless, miRBase is the standard source providing miRNA sequence information from 286 organisms (Release 22). Various classification algorithms have been used for miRNA precursor predictions. In this work, three of those classifiers, random forest, decision tree, and naïve Bayes, were trained and tested with the same datasets. Models of the random forest classifier produced higher performance scores of accuracy (Figures 3 and 4), F-measure, recall, precision, sensitivity, and specificity (Supplementary Figures 1 and 2), consistent with our previous research (Saçar Demirci and Allmer, 2017a).For ML analyses, some parameters explaining the dataset are required. There are various features proposed and used for miRNAs and these features can be grouped into structural, sequence-based, probability-based, and thermodynamic parameters. In earlier studies, we implemented hundreds of such features but we found that about 50 features are usually adequate for building an effective ML model (Saçar and Allmer, 2013a, 2013b). However, calculating such features is computationally expensive, especially for a genome-wide miRNA search. Moreover, the selection of informative features is an important step that has a large impact on the overall model performance (Yousef et al., 2016). Thus, an alternative approach like using 3D graphical representation of RNA secondary structures as features describing miRNAs seems like a promising method. 2D and 3D representations of RNA sequences create a data matrix based on the structural information. Although such representations have been used for measuring RNA similarities and classifying viruses (Yao et al., 2005; Li et al., 2012), they are rarely applied for pre-miRNA analysis (Fu et al., 2018). The workflow developed in this study is the first example of application of 3D representations of RNAs for ML-based miRNA prediction. The results presented here imply that when these features are used on a high quality dataset, they are sufficient for building a successful model for miRNA analysis. 

## Supplementary Material

Boxplots of classification performance measures when positive dataset was selected from MirGeneDB human miRNA entries: F-measure, recall, precision, sensitivity, specificity (from top to bottom).

Boxplots of classification performance measures when positive dataset was selected from MirGeneDB human miRNA entries: F-measure, recall, precision, sensitivity, specificity (from top to bottom).

Boxplots of classification performance measures when positive dataset was selected from MirGeneDB human miRNA entries: F-measure, recall, precision, sensitivity, specificity (from top to bottom).

Boxplots of classification performance measures when positive dataset was selected from MirGeneDB human miRNA entries: F-measure, recall, precision, sensitivity, specificity (from top to bottom).

Boxplots of classification performance measures when positive dataset was selected from MirGeneDB human miRNA entries: F-measure, recall, precision, sensitivity, specificity (from top to bottom).

Boxplots of classification performance measures when positive dataset was selected from MiRBase human miRNA entries: F-measure, recall, precision, sensitivity, specificity (from top to bottom).

Boxplots of classification performance measures when positive dataset was selected from MiRBase human miRNA entries: F-measure, recall, precision, sensitivity, specificity (from top to bottom).

Boxplots of classification performance measures when positive dataset was selected from MiRBase human miRNA entries: F-measure, recall, precision, sensitivity, specificity (from top to bottom).

Prediction performances on MirGeneDB data. Gray indicates miRNAs while red shows negatives. X-axis lists the acronyms of the organisms. Y-axis shows the number of precursors.
