# Supplementary figures and images for: MicroRNA prediction based on 3D graphical representation of RNA secondary structures
Source: Turk J Biol. 2019 Aug 5;43(4):274–80. doi: 10.3906/biy-1904-59 (PMC6713912; doi:10.3906/biy-1904-59)

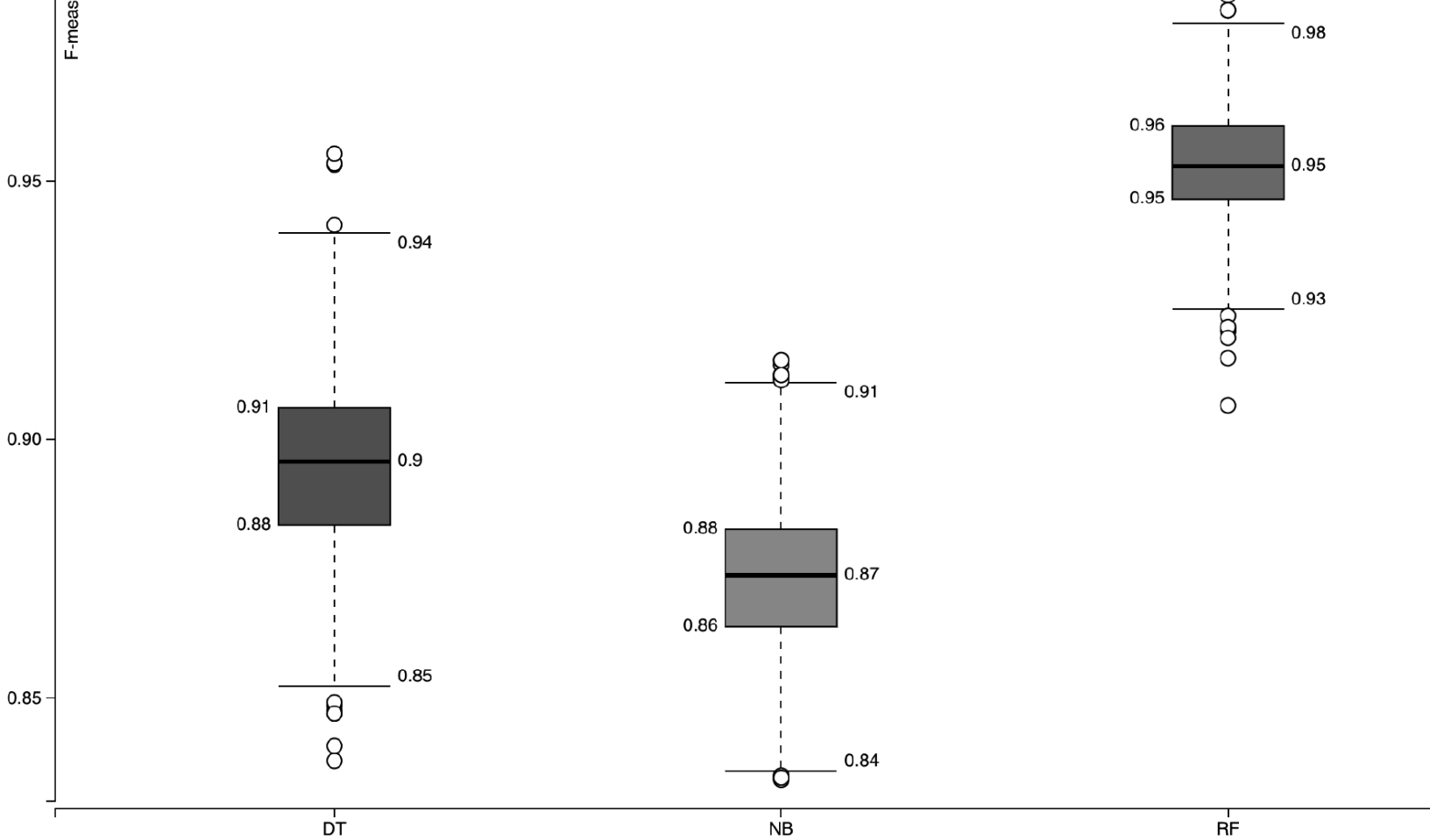

## MirGeneDB

human data for learning

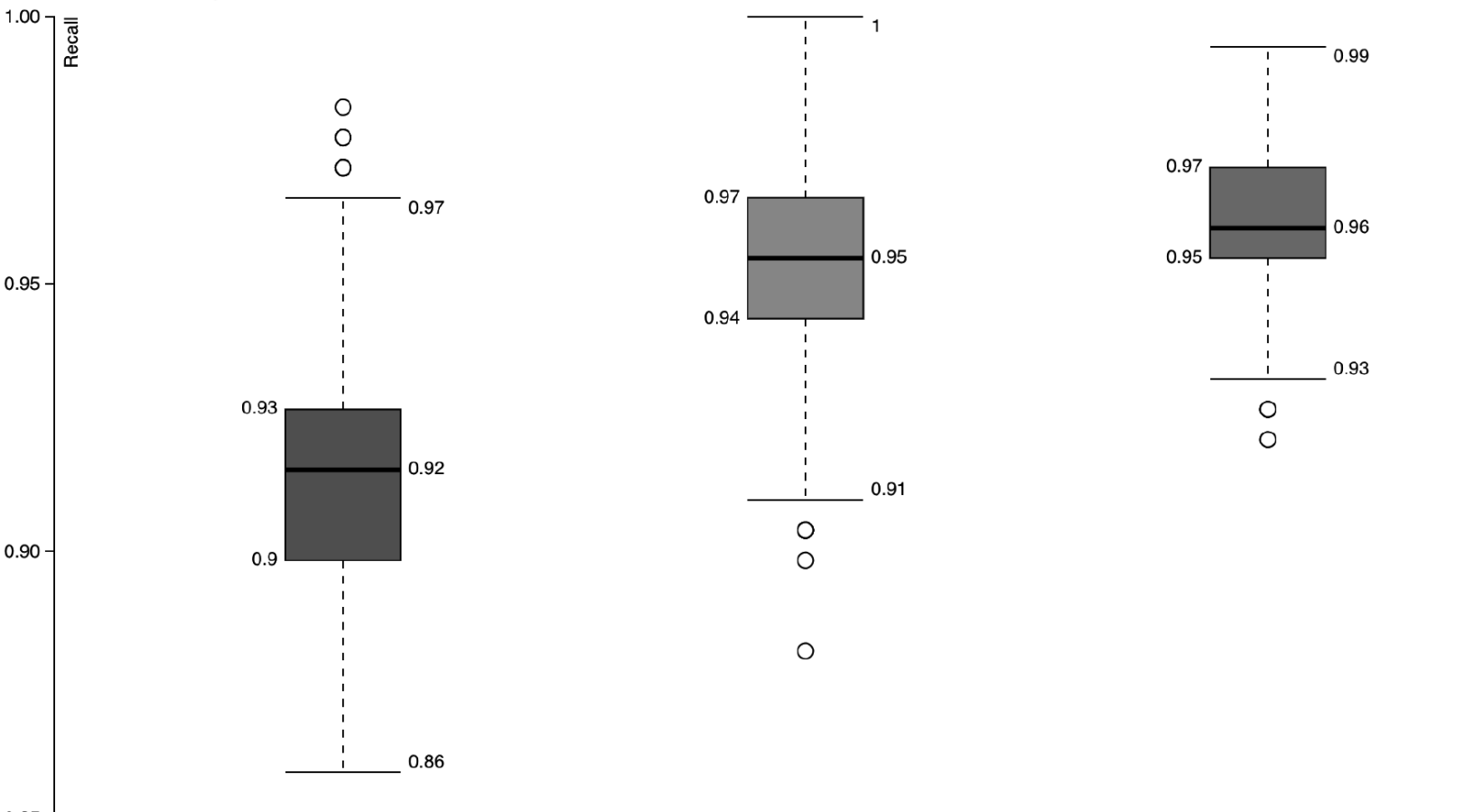

Supplement: Boxplots of classification performance measures when positive dataset was selected from MirGeneDB human miRNA entries: F-measure, recall, precision, sensitivity, specificity (from top to bottom). [file turkjbio-43-274-sup001.pdf]

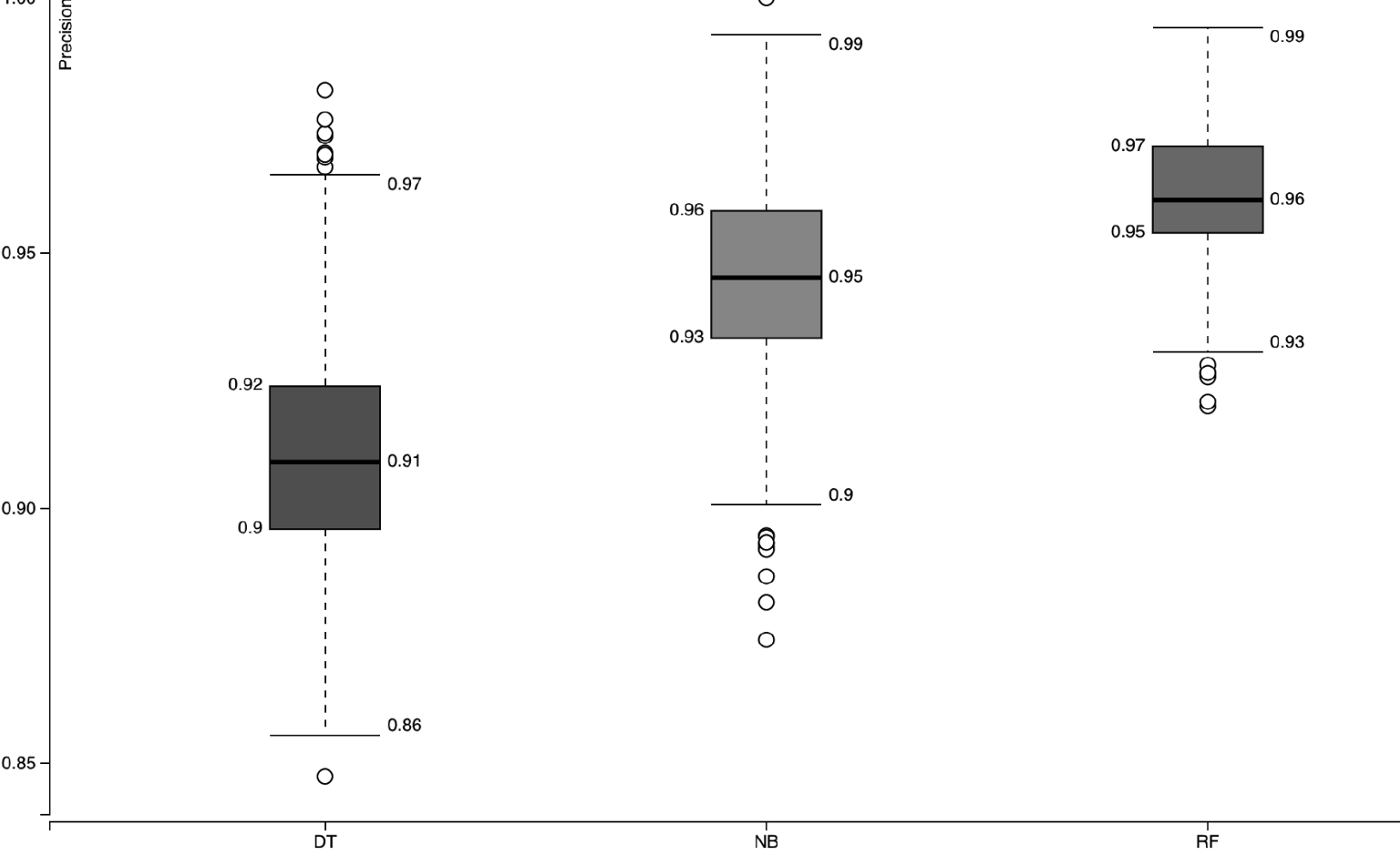

## MirGeneDB

human data for learning

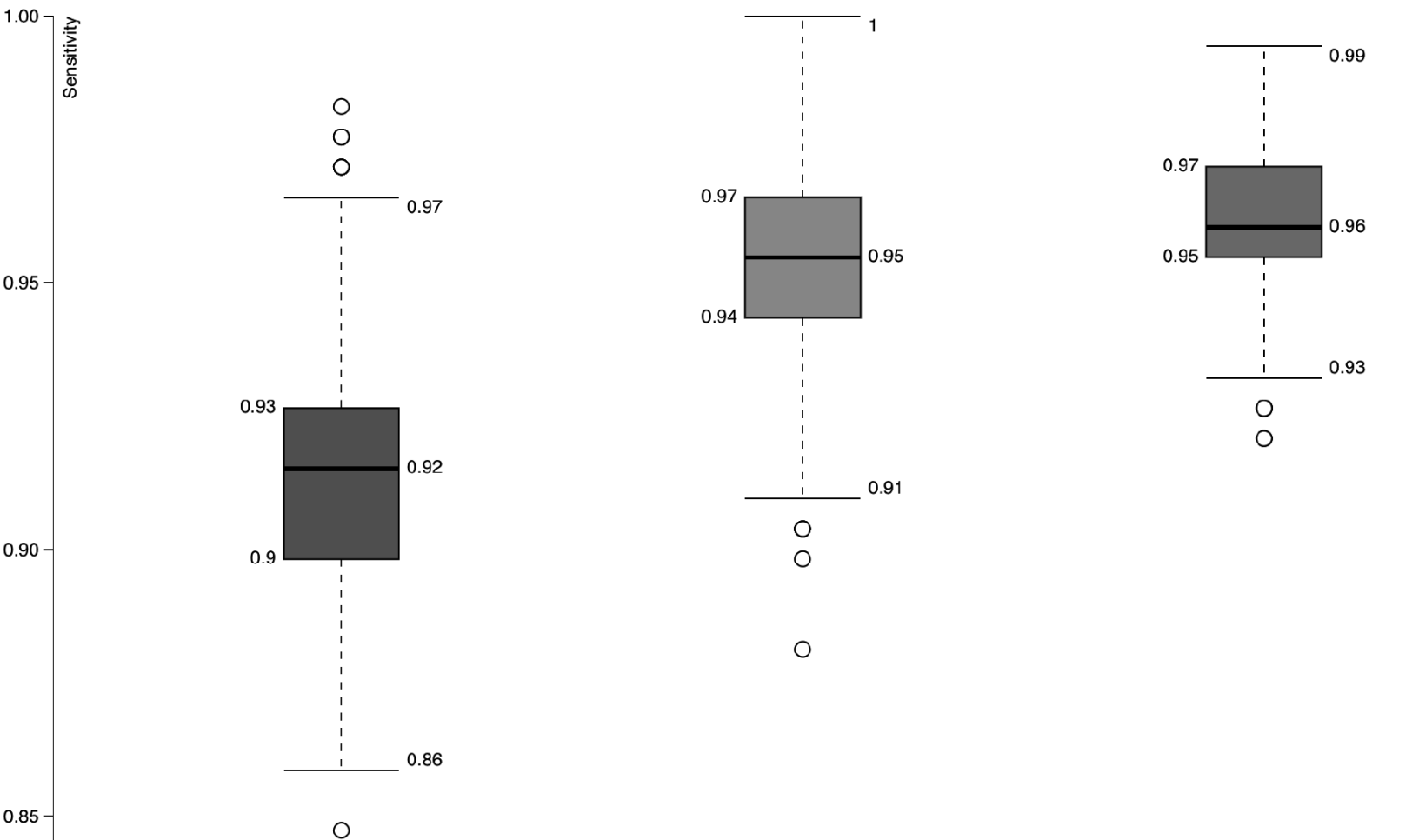

Supplement: Boxplots of classification performance measures when positive dataset was selected from MirGeneDB human miRNA entries: F-measure, recall, precision, sensitivity, specificity (from top to bottom). [file turkjbio-43-274-sup002.pdf]

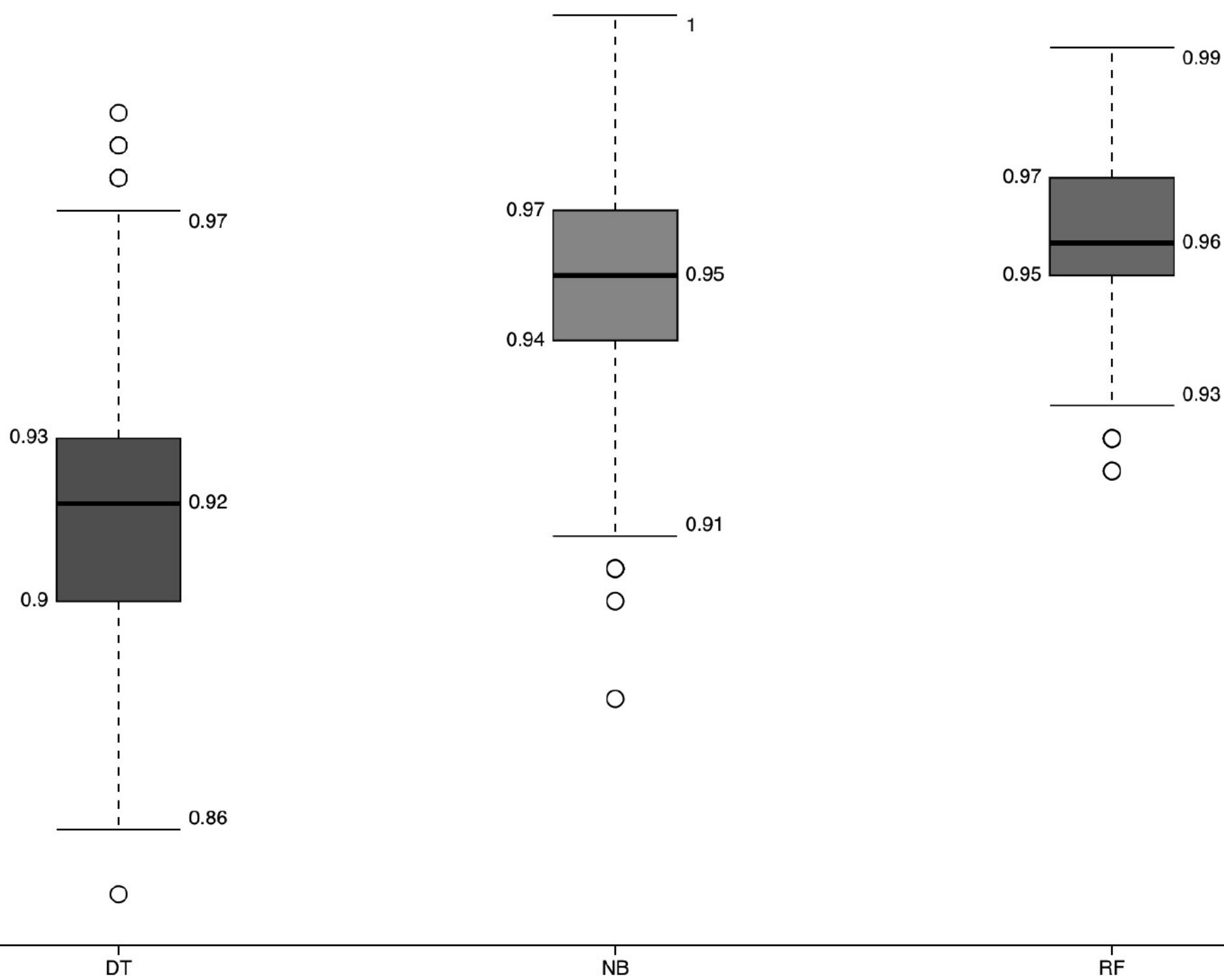

Supplement: Boxplots of classification performance measures when positive dataset was selected from MirGeneDB human miRNA entries: F-measure, recall, precision, sensitivity, specificity (from top to bottom). [file turkjbio-43-274-sup003.pdf]

# MiRBase

human data for learning

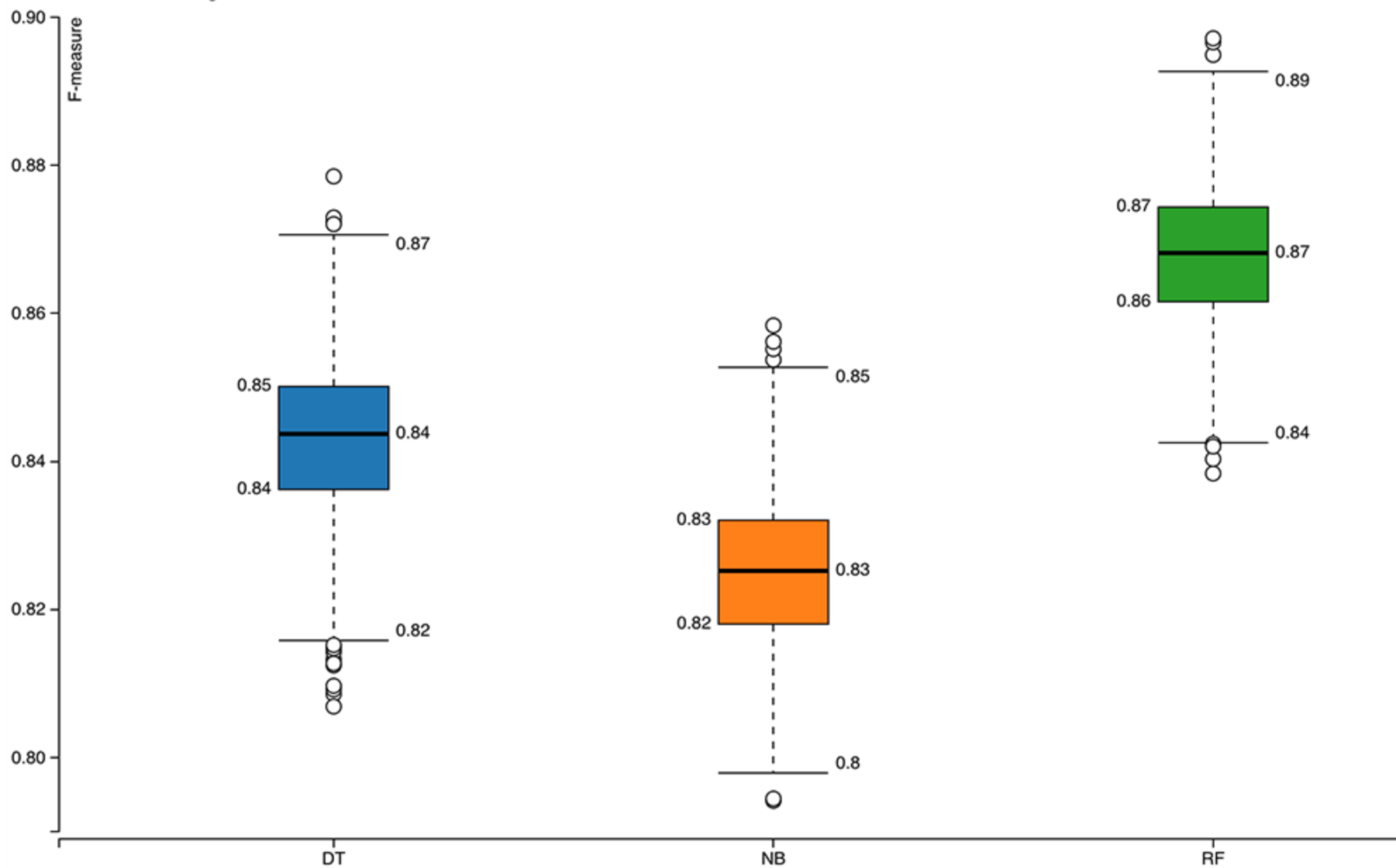

Supplement: Boxplots of classification performance measures when positive dataset was selected from MirGeneDB human miRNA entries: F-measure, recall, precision, sensitivity, specificity (from top to bottom). [file turkjbio-43-274-sup004.pdf]

# MiRBase

human data for learning

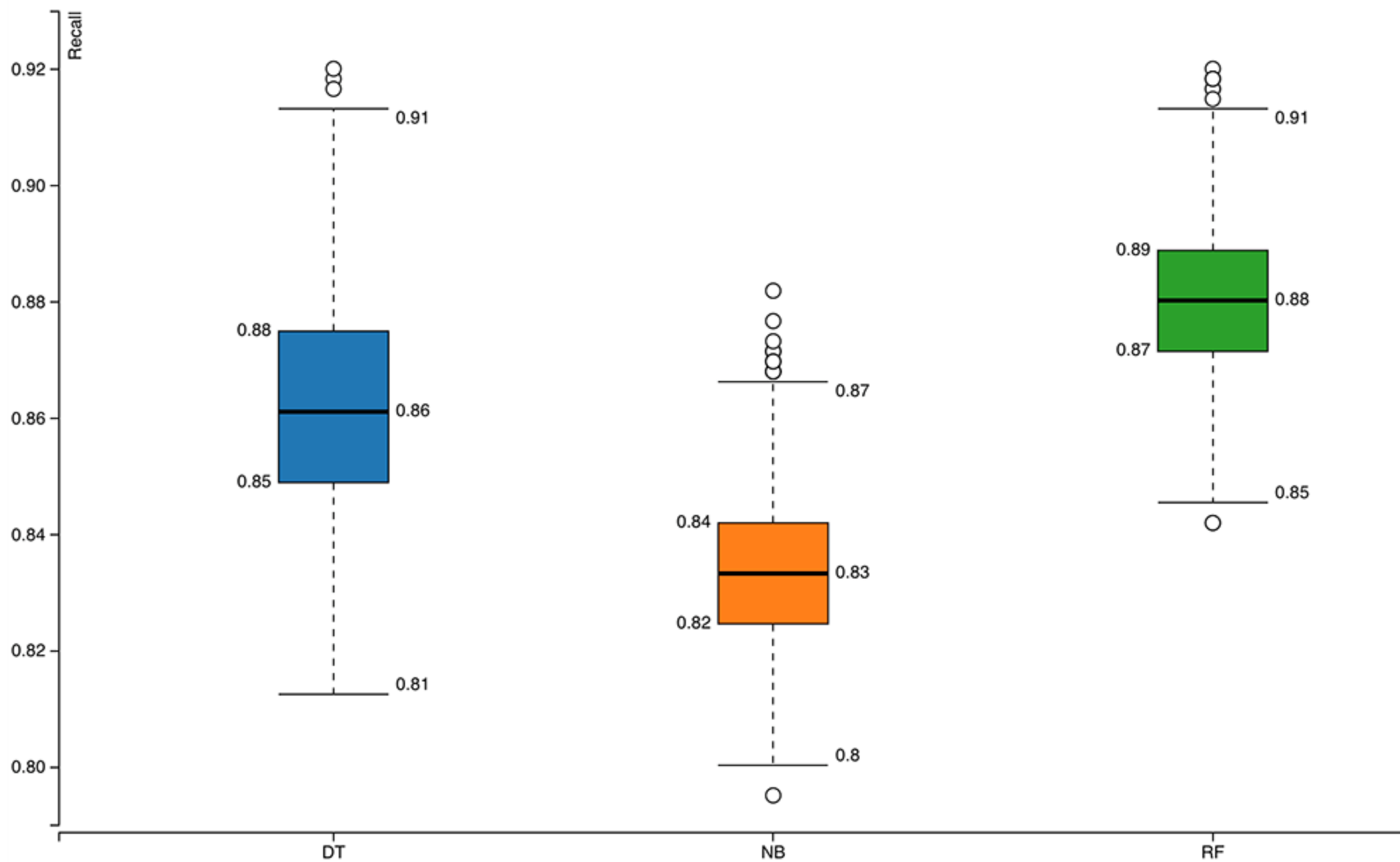

Supplement: Boxplots of classification performance measures when positive dataset was selected from MirGeneDB human miRNA entries: F-measure, recall, precision, sensitivity, specificity (from top to bottom). [file turkjbio-43-274-sup005.pdf]

# MiRBase

human data for learning

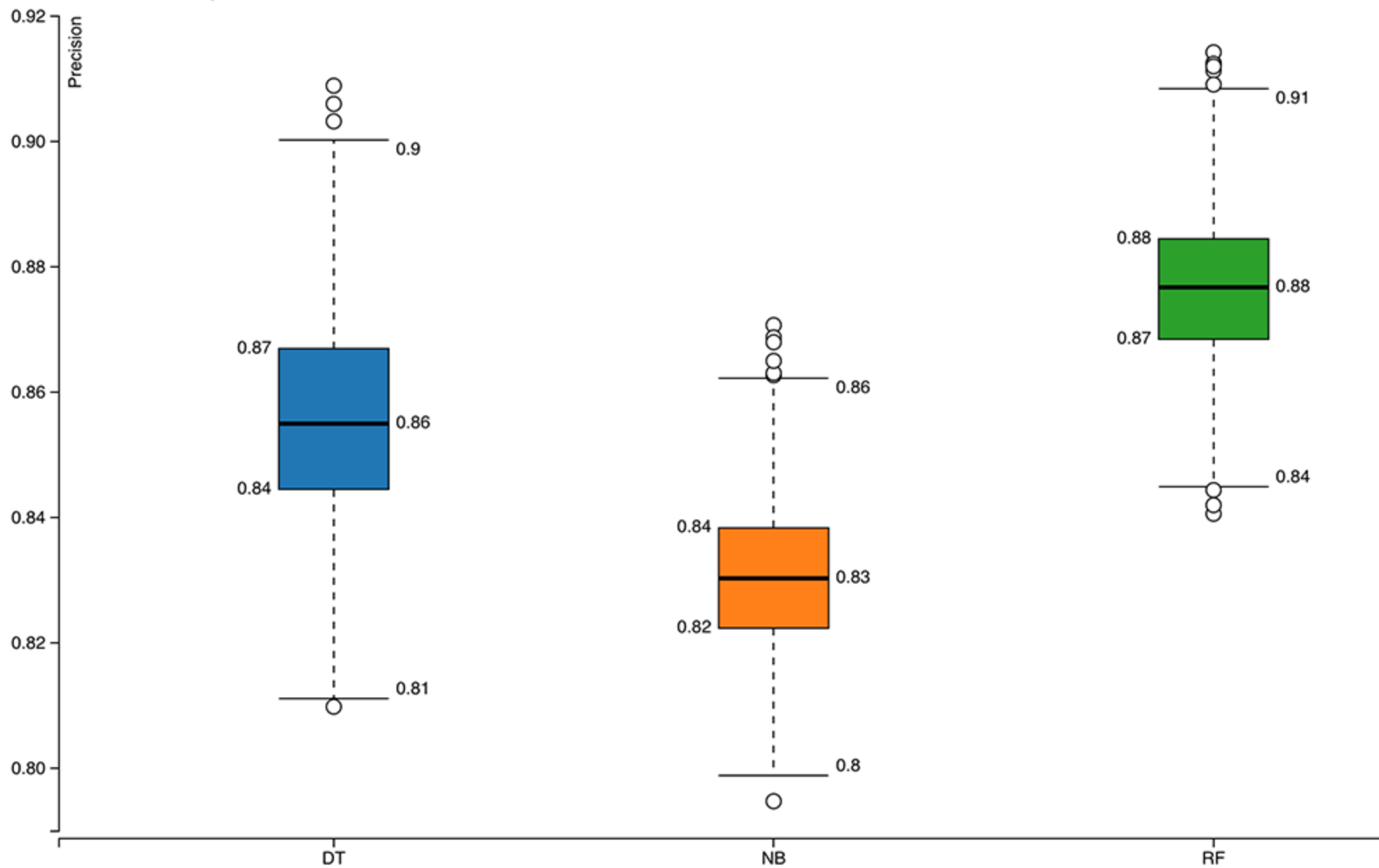

Supplement: Boxplots of classification performance measures when positive dataset was selected from MiRBase human miRNA entries: F-measure, recall, precision, sensitivity, specificity (from top to bottom). [file turkjbio-43-274-sup006.pdf]

# MiRBase

human data for learning

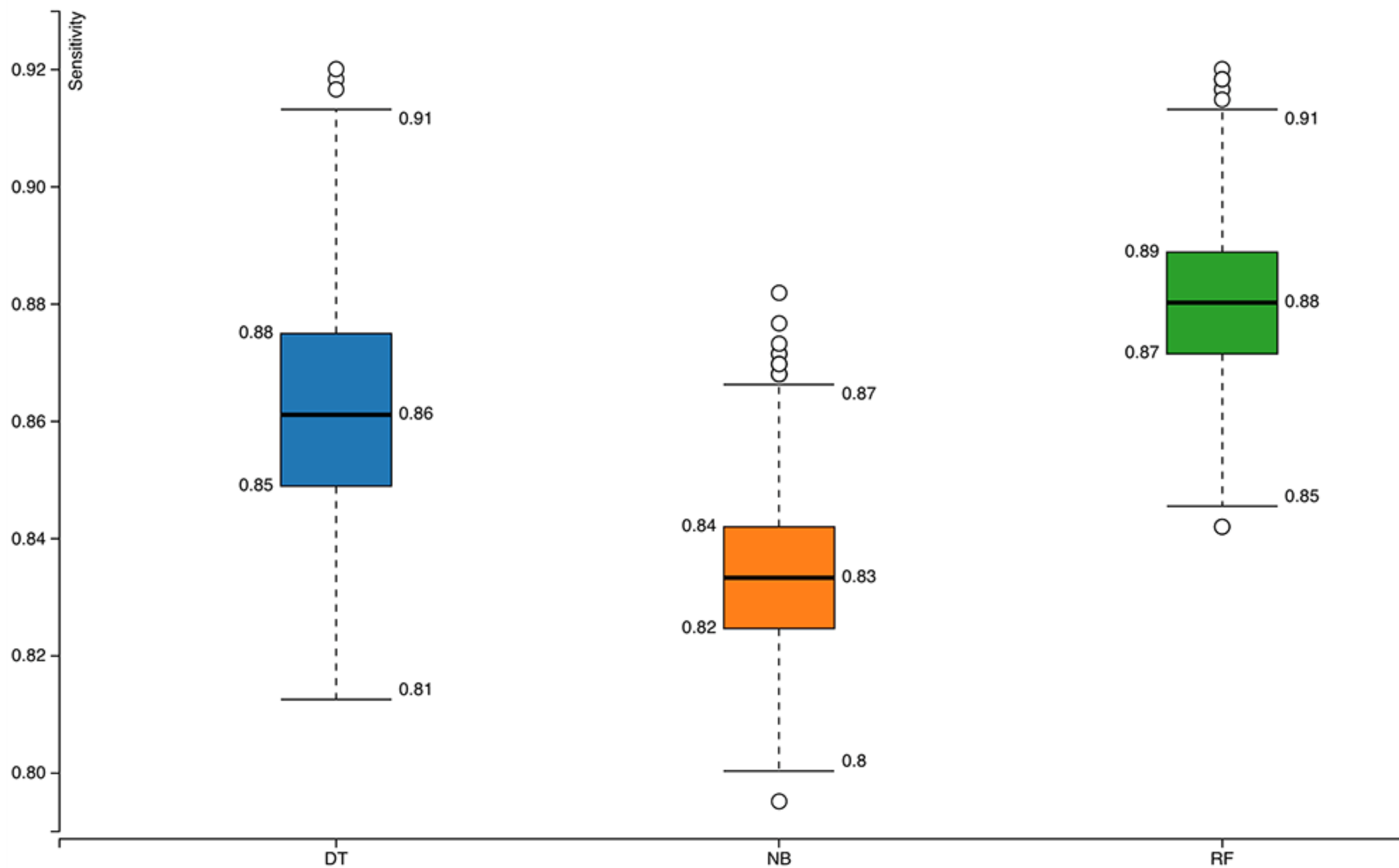

Supplement: Boxplots of classification performance measures when positive dataset was selected from MiRBase human miRNA entries: F-measure, recall, precision, sensitivity, specificity (from top to bottom). [file turkjbio-43-274-sup007.pdf]

# MiRBase

human data for learning

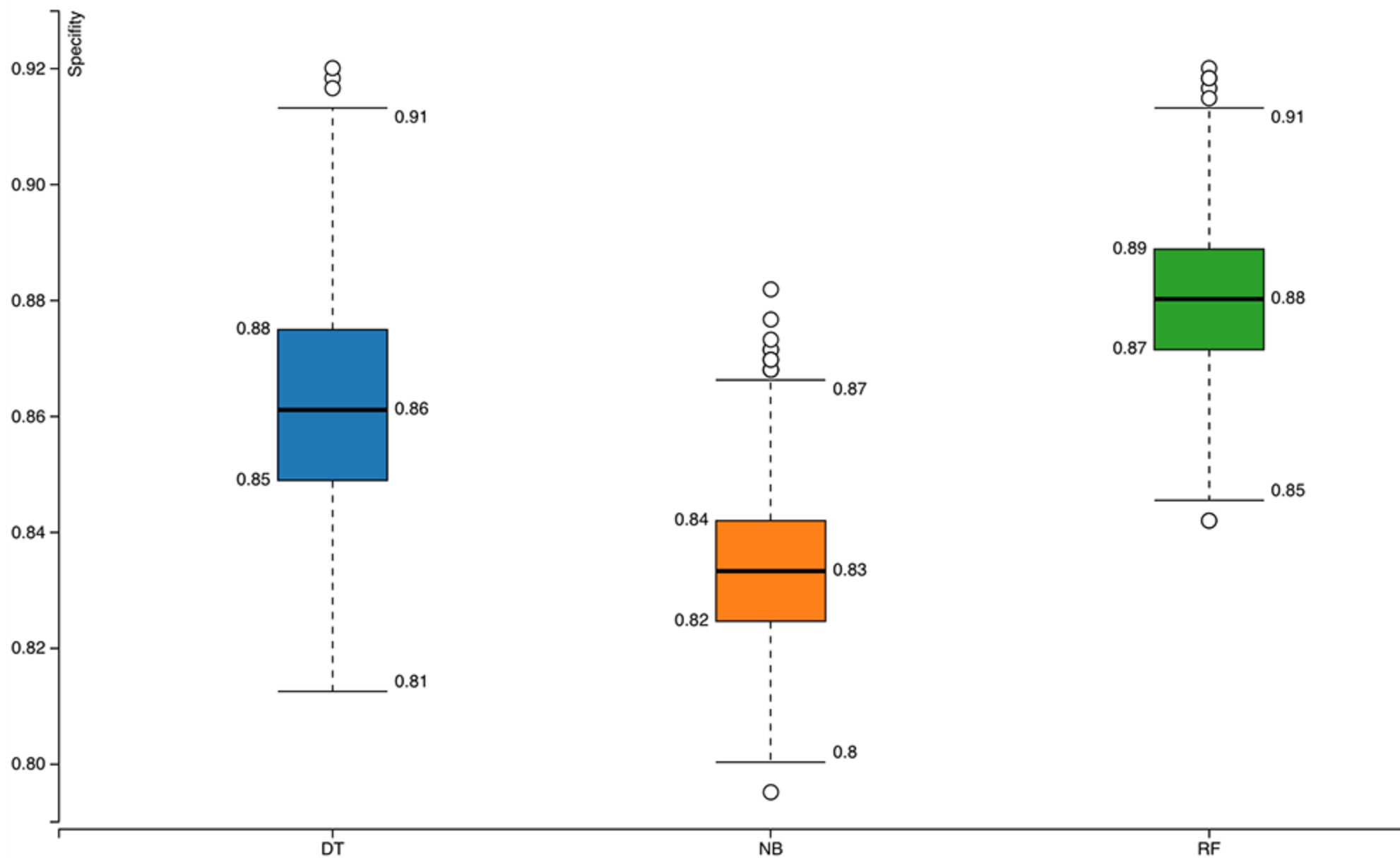

Supplement: Boxplots of classification performance measures when positive dataset was selected from MiRBase human miRNA entries: F-measure, recall, precision, sensitivity, specificity (from top to bottom). [file turkjbio-43-274-sup008.pdf]

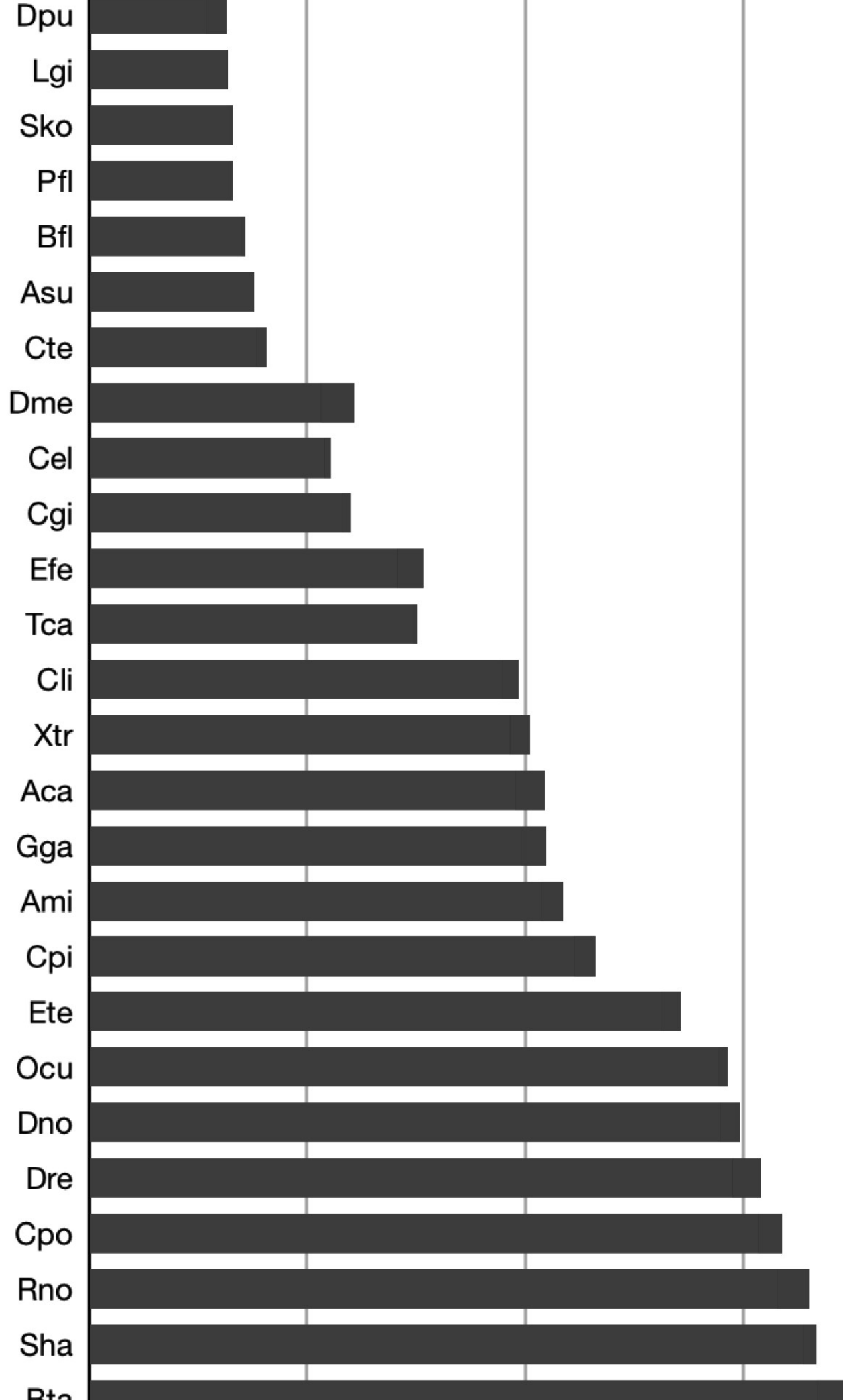

Supplement: Prediction performances on MirGeneDB data. Gray indicates miRNAs while red shows negatives. X-axis lists the acronyms of the organisms. Y-axis shows the number of precursors. [file turkjbio-43-274-sup009.pdf]
